# Vegetarian diets and risk of hospitalisation or death with diabetes in British adults: results from the EPIC-Oxford study

**DOI:** 10.1038/s41387-019-0074-0

**Published:** 2019-02-25

**Authors:** Keren Papier, Paul N. Appleby, Georgina K. Fensom, Anika Knuppel, Aurora Perez-Cornago, Julie A. Schmidt, Tammy Y. N. Tong, Timothy J. Key

**Affiliations:** Cancer Epidemiology Unit, Nuffield Department of Population Health, University of Oxford, Richard Doll Building, Old Road Campus, Oxford, UK

## Abstract

**Background:**

The global prevalence of diabetes is high and rapidly increasing. Some previous studies have found that vegetarians might have a lower risk of diabetes than non-vegetarians.

**Objective:**

We examined the association between vegetarianism and risk of hospitalisation or death with diabetes in a large, prospective cohort study of British adults.

**Methods:**

The analysed cohort included participants from the European Prospective Investigation into Cancer and Nutrition (EPIC)-Oxford study who were diabetes free at recruitment (1993–2001), with available dietary intake data at baseline, and linked hospital admissions and death data for diabetes over follow-up (*n* = 45,314). Participants were categorised as regular meat eaters (≥50 g per day: *n* = 15,181); low meat eaters (<50 g of meat per day: *n* = 7615); fish eaters (ate no meat but consumed fish: *n* = 7092); and vegetarians (ate no meat or fish, including vegans: *n* = 15,426). We used multivariable Cox proportional hazards models to assess associations between diet group and risk of diabetes.

**Results:**

Over a mean of 17.6 years of follow-up, 1224 incident cases of diabetes were recorded. Compared with regular meat eaters, the low meat eaters, fish eaters, and vegetarians were less likely to develop diabetes (hazard ratio (HR) = 0.63, 95% confidence interval (CI) 0.54–0.75; HR = 0.47, 95% CI 0.38–0.59; and HR = 0.63, 95% CI 0.54–0.74, respectively). These associations were substantially attenuated after adjusting for body mass index (BMI) (low meat eaters: HR = 0.78, 95% CI 0.66–0.92; fish eaters: HR = 0.64, 95% CI 0.51–0.80; and vegetarians: HR = 0.89, 95% CI 0.76–1.05).

**Conclusions:**

Low meat and non-meat eaters had a lower risk of diabetes, in part because of a lower BMI.

## Introduction

The number of people affected by diabetes globally is rapidly increasing, with estimates already surpassing 425 million in 2017 and projected to reach 629 million in 2045^1^. This imposes substantial economic burdens on healthcare systems^2^. In the United Kingdom, for example, around 10% of the 2011 health service budget was spent on treating diabetes^3^, giving the United Kingdom one of the highest per capita health expenditures for diabetes^1^.

The identification of modifiable risk factors is vital for the reduction of the growing diabetes epidemic. Diet is one such lifestyle factor that might play a key role in the prevention of diabetes. Evidence suggests that a diet abundant in whole grains, vegetables, fruits, dairy, legumes, and nuts may help lower the risk of diabetes, while one that is high in red and processed meat may increase it^4^. A recent meta-analysis found that consumption of an additional 100 g of red meat per day was associated with a 13% higher risk of diabetes and that an additional 50 g daily consumption of processed meat was associated with a 32% higher risk^5^.

Vegetarians, who omit meat and meat products, and, at least in Western countries, often consume more nuts and legumes than non-vegetarians^6,7^ might therefore be expected to have a lower risk of diabetes. Three prospective studies of Seventh-day Adventist adults in North America found that vegetarians had a lower risk of diabetes compared with those who consumed meat^8–10^. A more recent cohort study of Taiwanese Buddhists also reported that consuming a vegetarian diet was associated with a lower risk in diabetes occurrence^11^. To explore this further in a population-based, European cohort, we investigated the association between vegetarianism and diabetes in a large, population-based study of British adults participating in the European Prospective Investigation into Cancer and Nutrition (EPIC)-Oxford study, which includes a large number of vegetarians.

## Subjects and methods

### Study population

The EPIC-Oxford study is a prospective investigation of 65,411 men and women, established (as part of the EPIC Europe study) to examine health outcomes (diabetes included as a primary endpoint) in relation to diet in the United Kingdom. Participants were recruited through general practitioners (GPs) or by postal questionnaires between 1993 and 2001 and followed up with questionnaires approximately 15 years after the completion of the first survey. Participants recruited through participating GPs were 7421 men and women aged between 35 and 69 years who completed a full study questionnaire covering diet, lifestyle, health, and family medical history. Postal recruitment aimed to recruit vegetarian and vegan adults and other people with an interest in diet and health, and recruited 57,990 participants aged 20 or over years. Although the postal method was targeted at vegetarians, around 80% of meat eaters were also recruited via this method. The full study questionnaire was mailed to all surviving members of the Oxford Vegetarian Study and all members of the Vegetarian Society, who were invited to join and provide details of any friends or family who might also be interested in joining the study. A short questionnaire was also displayed in health food shops, in health food and vegetarian magazines, and sent out to all members of the Vegan Society; and the full questionnaire was later mailed to all those who returned this short questionnaire. All recruited participants who completed the full questionnaire were asked if they would be willing to provide access to their medical records for further information. The study protocol was approved by a Multicentre Research Ethics Committee (Scotland A Research Ethics Committee) and all participants provided written informed consent.

### Assessment of diabetes status

Diabetes status was ascertained through health record linkage. Participants were linked to National Health Service (NHS) Central Registers using their unique NHS number and other personal details from 1 April 1997 in England, 1 January 1981 in Scotland, and 1 January 1998 in Wales, until 31 March 2016. The data available included information on hospital admissions and deaths. Diagnoses identifying the reason for hospital admission and causes of death were coded according to the 9th and 10th revisions of the World Health Organization (WHO) International Classification of Diseases (ICD-9/10). Any diagnosis of diabetes (i.e., not necessarily the primary diagnosis) or mention of diabetes among the causes of death or contributory conditions was considered for this study, using ICD-9 code 250 and ICD-10 codes E10-E14.

### Assessment of diet and diet group

Dietary intake was assessed using the full questionnaire at baseline. Participants completed a validated 130-item semi-quantitative food frequency questionnaire (FFQ) reporting foods consumed over the past 12 months^12^. Participants were asked to indicate the frequency of consumption of each food with responses ranging from ‘never or less than once a month’ to ‘6 or more times daily’ and mean daily nutrient intakes were calculated by multiplying frequencies of consumption by standard portion sizes^13^ and using food codes^14^. Responses to the 11 questions on meat on the FFQ were used to calculate total meat intake. In addition to the FFQ, participants were asked to report whether they consumed any of the following: meat, fish, dairy products, and eggs. Responses to the FFQ and the four questions were used to categorise participants into 5 diet groups: regular meat eaters (participants who consumed ≥50 g of meat daily); low meat eaters (participants who consumed <50 g of meat daily); fish eaters (participants who consumed fish but did not consume meat); vegetarians (participants who did not consume meat or fish but did consume dairy products, or eggs); and vegans (participants who did not consume fish, meat, dairy products, or eggs). For the main analysis, vegetarians and vegans were combined into one dietary group due to small numbers in the vegan group, but vegans were also assessed separately in a supplementary analysis.

### Assessment of covariates

Potential confounders recorded at the baseline survey included socio-economic status (Townsend deprivation index^15^ (quartiles, unknown) and educational level (no qualifications, basic secondary (e.g., O level), higher secondary (e.g., A level), university degree, unknown)); demographic factors (age (continuous)); lifestyle factors (smoking (never, former, light, heavy), alcohol consumption (<1 g, 1–7 g, 8–15 g, ≥16 g/day), and physical activity^16^ (inactive, low activity, moderately active, very active, unknown)); and other factors including ethnicity (white, other, unknown) and measured or self-reported height and weight^17^. Body mass index (BMI) was calculated by dividing weight in kilograms by the square of height in metres and categorised as <22.5, 22.5–24.4, 24.5–26.4, 26.5–28.4, 28.5–30.4, 30.5–34.9, and ≥35.0 kg/m^2^.

### Exclusions

Participants were excluded if they resided outside England, Scotland, or Wales (*n* = 945), if they did not have an NHS number, hospital admissions or death data, or if they could not be traced by the NHS (*n* = 34) (Supplemental Fig. 1). Participants who only completed the short questionnaire were also excluded (*n* = 7619), as were those who were younger than 20 years or 90 years or over (*n* = 59) at recruitment; and those who did not have any follow-up data (*n* = 364). Participants reporting prevalent malignant cancers (except non-melanoma skin cancer) (*n* = 1 967) were also excluded along with those self-reporting prior diabetes (*n* = 5303), heart attack (*n* = 476), or stroke (*n* = 180). All participants with unreliable dietary data were excluded (*n* = 1293) as were those with an unknown BMI (*n* = 1630) or smoking status (*n* = 227). Accordingly, the analysed cohort included 45,314 participants.

### Statistical analysis

Baseline characteristics of eligible participants were compared across diet groups. We used Cox proportional hazards models to assess the associations between diet groups and incidence of diabetes. Age was used as the underlying time variable with person years calculated from age at recruitment or the beginning of the hospital record data, whichever was later, until the age at diagnosis, death, or administrative censoring. All analyses were stratified by sex, method of recruitment (GP or postal), and region of residence (7 regions across the United Kingdom). In model 1 we estimated hazard ratios (HRs) and 95% confidence intervals (CIs) adjusted for age, ethnicity, education level, Townsend deprivation index, smoking, alcohol consumption, and physical activity level. In model 2 we further adjusted for BMI. We then assessed whether selected food and nutrient intakes (energy, carbohydrates, starch, fibre, protein, and fat) were associated with diabetes risk to identify potential nutritional confounders or mediators of the association of diet group with diabetes; we found that inclusion of these factors in our models did not change the association between diet groups and diabetes risk (results not shown) and therefore we did not include these in our final analyses. Likelihood ratio tests were used to assess statistical differences in risk between diet groups for all models. Because diabetes risk has been found to vary between different types of meatless diets^10^ we also tested for pairwise differences in the risk of diabetes between the fish eaters and the vegetarians using a post hoc test.

The relationships between meat intake and diabetes may vary by personal or socio-demographic characteristics^18^. Therefore, we assessed heterogeneity in the association between diet group and diabetes by sex, age, smoking status, educational level, and BMI by adding appropriate interaction terms in the Cox models and testing for statistical significance of interaction using likelihood ratio tests. We also assessed associations between diabetes and BMI, subdivided by diet group. We additionally assessed mean change in age- and sex-adjusted BMI by baseline diet group for participants who completed both the baseline and the 15-year EPIC-Oxford follow-up survey. All analyses were carried out using Stata (version 15.0). All statistical tests were two-sided with *p* values < 0.05 considered statistically significant.

## Results

The baseline characteristics of participants by diet groups are shown in Table 1. Approximately two-thirds of participants were categorised as low meat or non-meat eaters. On average, low meat and non-meat eaters were younger, had a lower socio-economic status, higher education level, and reported lower levels of smoking and alcohol consumption than regular meat eaters. Compared with regular meat eaters, physical activity levels were higher and BMI was lower in low meat and non-meat eaters. Consumption of cheese, pulses, nuts, fruit, vegetables, plant protein, total and intrinsic sugar, carbohydrate, fibre, and starch was higher among low and non-meat eaters compared with regular meat eaters, while the opposite was true for each of total, red, and processed meat, animal milk, total energy, added sugar, total and animal protein, and total fat, monounsaturated fat, and saturated fat.

**Table 1:** Baseline characteristics of 45 314 EPIC-Oxford participants by diet group

Characteristics	Regular meat eaters ≥50 g/day	Low meat eaters <50 g/day	Fish eaters	Vegetarians
Mean (SD), or *n* (%)	*N*=15,181	*N*=7615	*N*=7092	*N*=15,426
Socio-demographic				
Age, years^a^	49.5 (12.9)	47.3 (13.5)	41.8 (12.7)	39.3 (13.0)
Sex, women (%)	11,133 (73)	6041 (79)	5822 (82)	11,581 (75)
Top socio-economic quartile (%)^b,c^	3943 (30)	1614 (25)	1336 (22)	2876 (21)
Higher education (%)^b^	3951 (28)	2988 (42)	3148 (46)	6382 (43)
Lifestyle and health				
Current smokers (%)	1913 (13)	835 (11)	718 (10)	1592 (10)
Alcohol consumption, g/day	10.7 (13.5)	9.2 (11.5)	10.2 (12.4)	9.3 (12.8)
Moderate/high physical activity (%)^b^	4009 (30)	2379 (36)	2560 (41)	5579 (40)
Body mass index (kg/m^2^)	24.6 (3.9)	23.4 (3.5)	22.9 (3.4)	22.8 (3.4)
Diet				
Total meat & meat products (g/day)	99.8 (41.6)	28.1 (12.8)	–	–
Red & processed meat(g/day)	65.4 (38.1)	18.8 (10.3)	–	–
Total fish & fish products (g/day)	43.8 (28.8)	38.4 (29.3)	38.6 (33.3)	0.6 (5.1)
Animal milk (ml/day)^b^	334.9 (183.1)	299.2 (185.2)	273.7 (189.0)	232.4 (207.8)
Total dairy cheese (g/day)	19.7 (17.5)	22.8 (20.4)	27.4 (24.1)	26.8 (25.4)
Total beans, pulses & soya products^d^ (g/day)	25.8 (25.9)	35.4 (35.9)	57.9 (44.1)	74.7 (56.7)
Total nuts and nut butters (g/day)	4.0 (7.8)	6.2 (11.3)	7.9 (11.6)	10.6 (16.1)
Total fresh fruit (g/day)	248.5 (185.9)	294.7 (237.9)	289.8 (226.3)	284.1 (240.0)
Total fresh vegetables (g/day)	248.1 (123.5)	257.6 (145.9)	285.5 (147.0)	293.7 (162.4)
Total energy (kJ/day)	8627 (2225)	7628 (2137)	7926 (2199)	7821(2232)
Carbohydrate (%E)	46.8 (5.8)	50.5 (6.3)	50.9 (6.5)	52.8 (6.8)
Total sugars (%E)	23.6 (5.3)	25.8 (6.2)	25.0 (6.2)	25.3 (6.6)
Added sugars (%E)	9.1 (4.6)	8.2 (4.7)	7.9 (4.2)	8.5 (4.6)
Intrinsic sugars (%E)	14.6 (5.3)	17.6 (6.4)	17.1 (6.4)	16.8 (6.8)
Starch (%E)	23.3 (4.7)	24.9 (5.4)	26.2 (5.5)	27.9 (5.9)
Total fibre (g)	18.4 (6.4)	19.4 (7.4)	21.1 (7.4)	22.1 (8.0)
Protein (%E)	17.6 (3.0)	15.5 (2.5)	14.6 (2.3)	13.6 (2.1)
Protein from animal products (%E)	10.9 (2.9)	7.7 (2.6)	5.8 (2.4)	3.7 (2.3)
Protein from plant products (%E)	5.8 (1.1)	6.7 (1.3)	7.2 (1.4)	7.8 (1.7)
Total fat (%E)	32.0 (5.6)	30.5 (6.2)	30.8 (6.3)	30.2 (6.6)
Saturated fat (%E)	11.8 (3.2)	10.9 (3.4)	10.6 (3.3)	10.2 (3.5)
Monounsaturated fat (%E)	11.0 (2.2)	10.0 (2.4)	9.9 (2.4)	9.7 (2.6)
Polyunsaturated fat (%E)	6.3 (1.8)	6.4 (2.2)	7.0 (2.2)	7.1 (2.5)
EPA (%E)	0.033 (0.024)	0.033 (0.027)	0.032 (0.030)	0.002 (0.007)
DHA (%E)	0.059 (0.043)	0.059 (0.048)	0.057 (0.053)	0.004 (0.012)

The *x*^2^ test was used to compare the distribution between diet groups for all categorical variables. Analysis of variance (ANOVA) was used to compare the means between the diet groups. The *P*-heterogeneity between diet groups was   <0.001 for all variables

*EPIC* European Prospective Investigation into Cancer and Nutrition, *%E* percent energy, *EPA* eicosapentaenoic acid, *DHA* docosahexaenoic acid

^a^Age at recruitment or hospital episode statistics start date

^b^Numbers may not add to total sample size due to missing responses

^c^Based on Townsend deprivation index

^d^Excluding soya milk

**Fig. 1:**
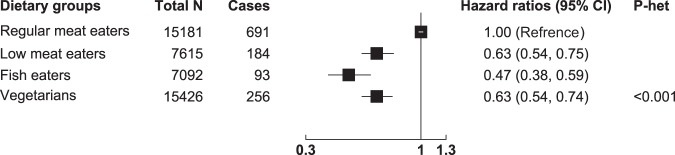
Associations between diet group and diabetes incidence in 45,314 EPIC-Oxford participants. Regular meat eaters were defined as participants who consumed ≥50 g of meat per day and low meat eaters were defined as participants who consumed <50 g of meat per day. Model 1: Cox regression analysis stratified by sex, method of recruitment, region of residence, and adjusted for age, education, Townsend deprivation index, ethnicity, smoking, alcohol intake, and physical activity. *P*-heterogeneity represents significant heterogeneity in risk between diet groups based on Wald test statistics. EPIC European Prospective Investigation into Cancer and Nutrition

Over a mean of 17.6 years of follow-up, there were 1224 incident cases of diabetes (11 of which were first noted at death). Compared with the regular meat eaters, the low meat eaters (HR = 0.63, 95% CI 0.54–0.75), fish eaters (HR = 0.47, 95% CI 0.38–0.59), and vegetarians (HR = 0.63, 95% CI 0.54–0.74) all had a lower risk of developing diabetes (likelihood ratio *Χ*^2^ test statistic = 70.50, *p*-heterogeneity 3.34 × 10^−15^) (Fig. 1); addition of BMI to the model (model 2) substantially attenuated the HR estimates for all three groups (low meat eaters: HR = 0.78, 95% CI 0.66–0.92; fish eaters: HR = 0.64, 95% CI 0.51–0.80; and vegetarians: HR = 0.89, 95% CI 0.76–1.05, likelihood ratio *Χ*^2^ test statistic = 19.57, *p*-heterogeneity 2.00 × 10^−04^). Pairwise comparisons between fish eaters and vegetarians showed that diabetes risk was significantly lower in the fish eaters compared with the vegetarians (model 1: HR = 0.74, 95% CI 0.59–0.94, *p*-heterogeneity = 0.015; model 2: HR = 0.72, 95% CI 0.56–0.91, *p*-heterogeneity = 0.007). When the vegetarians and vegans were assessed separately, both groups had a lower risk of developing diabetes compared with regular meat eaters (vegetarians: HR = 0.65, 95% CI 0.55–0.76; vegans: HR = 0.53, 95% CI 0.36–0.79) in model 1 (not adjusted for BMI), Supplemental Table 1.

Examination of the associations between sex-specific quintiles of nutrient intake and diabetes showed that overall energy, carbohydrate, starch, protein intake, and fat intake were not associated with diabetes risk (Supplemental Table 2). Higher intake of added sugars was associated with a higher risk of diabetes (HR = 1.38, 95% CI 1.14–1.67 when comparing the highest and lowest quintiles of intake, *p*_trend_ < 0.001), whereas higher consumption of intrinsic sugars, dietary fibre, and plant protein were each associated with a lower risk of diabetes (highest versus lowest quintile: HR = 0.71, 95% CI 0.58–0.85, *p*_trend_ < 0.001; HR = 0.81, 95% CI 0.67–0.99, *p*_trend_ = 0.02; and HR = 0.72, 95% CI 0.59–0.88, *p*_trend_ < 0.001, respectively).

**Fig. 2:**
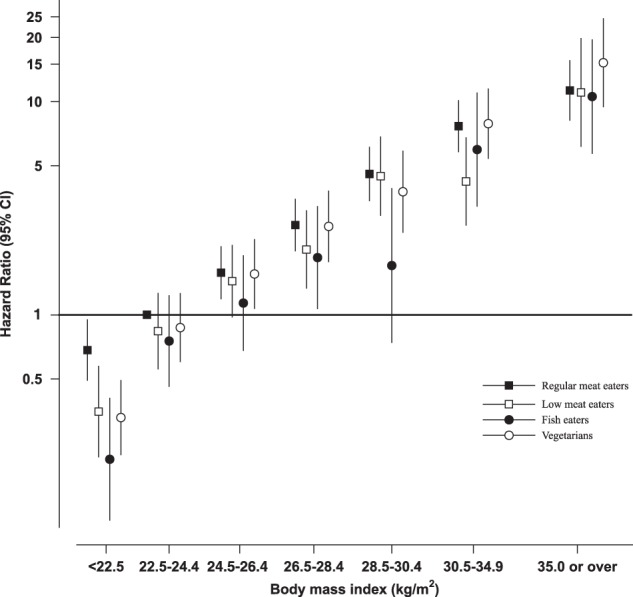
Hazard ratios for diabetes in EPIC-Oxford men and women by body mass index and diet group. Black squares indicate regular meat eaters, white squares low meat eaters, black circles fish eaters, and white circles vegetarians. Cox regression analysis stratified by sex, method of recruitment, region of residence, and adjusted for age, education, Townsend deprivation index, ethnicity, smoking, alcohol intake, and physical activity. Hazard ratios are relative to regular meat eaters with a body mass index reference level of 22.5–24.4 kg/m^2^. CI confidence interval, EPIC European Prospective Investigation into Cancer and Nutrition

The subgroup analyses showed some evidence of heterogeneity of risk by diet group for BMI (*p* = 0.05) but not by age, sex, smoking status, or educational level (*p* > 0.05) (Table 2). Figure 2 shows the association between BMI and diabetes divided by the four diet groups. In all four diet groups the risk of diabetes was strongly associated with BMI, with generally small differences in risk between diet groups within each category of BMI.

**Table 2:** Prospective associations between diet group and diabetes by different subgroups

	Hazard ratios^a^, 95% CI for subgroups			*P* value^b^
**Sex**	**Men**	**Women**		*P* = 0.11
*N* Cases/non-cases	405/10,332	819/33,758		
Regular meat eaters	Ref	Ref		
Low meat eaters	0.93 (0.69, 1.25)	0.74 (0.60, 0.90)		
Fish eaters	0.82 (0.54, 1.23)	0.58 (0.44, 0.76)		
Vegetarians	0.83 (0.61, 1.11)	0.94 (0.77, 1.14)		
*P*-heterogeneity^c^	0.57	0.002		
**Age at recruitment**	**<** **35 Years**	**35–49 Years**	**≥** **50 Years**	*P* = 0.50
*N* Cases/non-cases	87/12,472	340/16,889	797/14,729	
Regular meat eaters	Ref	Ref	Ref	
Low meat eaters	0.93 (0.43, 2.03)	0.82 (0.57, 1.16)	0.75 (0.62, 0.92)	
Fish eaters	0.65 (0.30, 1.42)	0.73 (0.48, 1.10)	0.64 (0.48, 0.86)	
Vegetarians	1.05 (0.61, 1.81)	1.16 (0.88, 1.52)	0.75 (0.60, 0.95)	
*P*-heterogeneity^c^	0.60	0.08	<0.001	
**Body mass index**	**<25** **kg/m**^**2**^	**≥25 to** **<30** **kg/m**^**2**^	**≥30** **kg/m**^**2**^	*P* = 0.05
*N* Cases/non-cases	367/32,657	482/9272	375/2161	
Regular meat eaters	Ref	Ref	Ref	
Low meat eaters	0.67 (0.49, 0.90)	0.84 (0.65, 1.08)	0.75 (0.54, 1.06)	
Fish eaters	0.56 (0.39, 0.80)	0.54 (0.37, 0.80)	0.88 (0.57,1.36)	
Vegetarians	0.68 (0.51, 0.89)	0.87 (0.67, 1.14)	1.13 (0.84, 1.52)	
*P*-heterogeneity^c^	0.001	0.02	0.20	
**Smoking status**	**Never**	**Former**	**Current**	*P* = 0.12
*N* Cases/non-cases	640/26,754	423/12,439	161/4,897	
Regular meat eaters	Ref	Ref	Ref	
Low meat eaters	0.70 (0.55, 0.88)	0.98 (0.75, 1.29)	0.65 (0.38, 1.12)	
Fish eaters	0.68 (0.50, 0.92)	0.68 (0.46, 1.00)	0.43 (0.18, 1.03)	
Vegetarians	0.88 (0.71, 1.10)	0.88 (0.66, 1.16)	0.84 (0.50, 1.42)	
*P*-heterogeneity^c^	0.005	0.23	0.15	
**Education level**	**Age 16**	**Age 18**	**Degree**	*P* = 0.41
*N* Cases/non-cases	600/14,864	250/10,330	253/16,216	
Regular meat eaters	Ref	Ref	Ref	
Low meat eaters	0.93 (0.74, 1.17)	0.53 (0.35, 0.80)	0.64 (0.44, 0.95)	
Fish eaters	0.61 (0.43, 0.87)	0.62 (0.38, 1.03)	0.66 (0.44, 1.01)	
Vegetarians	0.92 (0.72, 1.18)	0.87 (0.62, 1.23)	0.79 (0.57, 1.09)	
*P*-heterogeneity^c^	0.06	0.02	0.07	

^a^Cox regression analysis stratified by sex, method of recruitment, region of residence, and adjusted for age, education, Townsend deprivation index, ethnicity, smoking, alcohol intake, physical activity and body mass index

^b^Represents test for statistical significance of interaction across strata using likelihood ratio tests

^c^Represents significance of heterogeneity in risk between diet groups using Wald tests

The age- and sex-adjusted mean increase in BMI between the baseline and the 15-year follow-up survey was highest in regular meat eaters (mean increase = 1.78 kg/m^2^, 95% CI 1.71–1.85), followed by vegetarians (mean increase = 1.59 kg/m^2^, 95% CI 1.53–1.65), low meat eaters (mean increase = 1.45 kg/m^2^, 95% CI 1.36–1.54), and fish eaters (mean increase = 1.39 kg/m^2^, 95% CI 1.30–1.47) (*p*-heterogeneity < 0.001).

## Discussion

This is the largest prospective study of vegetarianism and diabetes risk in a population-based cohort. Our results indicate that low and non-meat diets are associated with a lower risk of diabetes in this population. Lower BMI in low and non-meat eaters appears to be at least partially responsible for this protective association.

Our findings confirm results from previous cohort studies that found that diabetes risk was 40–50% lower in those consuming a low^19^ or meat-free diet^10,11^. In the current study, the vegetarians were 37% less likely to develop diabetes compared with the regular meat eaters before BMI adjustment and 11% less likely after BMI adjustment (not statistically significant). In the Adventist Health Study 2 (AHS-2), lacto-ovo vegetarians were 54% less likely to develop diabetes before BMI adjustment and 38% less likely after BMI adjustment. In the The Tzu Chi Health Study (TCHS), vegetarians were 46% less likely to develop diabetes before BMI adjustment and 35% less likely after BMI adjustment. Likewise, the low meat eaters in our study had a 37% lower risk of diabetes compared with the regular meat eaters before BMI adjustment and a 22% lower risk after BMI adjustment. A similar risk reduction was reported in a US study of nurses and health professionals consuming a semi-vegetarian (or ‘plant-based diet') who had a 45% lower risk of diabetes before BMI adjustment and 34% risk after adjustment^19^. Compared with regular meat consumers, fish eaters in the current study had the lowest risk of developing diabetes (53% before BMI adjustment; 36% after BMI adjustment); a somewhat similar magnitude of risk reduction for fish eaters was observed in a previous study from AHS-2 (40% before BMI adjustment; 21% and not statistically significant after BMI adjustment)^10^.

As in previous studies^20,21^, higher BMI was strongly associated with diabetes risk in this cohort and the magnitude of the increase in risk was broadly similar across all four diet groups. There was, however, some evidence of heterogeneity in risks between the diet groups when subdivided by categories of BMI, with the biggest differences in risk observed in the lowest BMI category (<25 kg/m^2^). It is possible that in people who are obese the adverse effect of obesity on diabetes risk overrides any protection of diet composition^20^. Obesity is a well-established cause of diabetes; adipose cells secrete hormones and adipokines that increase insulin resistance and the subsequent risk of diabetes^22^.

It is possible that some of the reduced risk of diabetes in the low and non-meat consumers might be independent of BMI and might be attributed to the reduction or avoidance of certain exposures from meat and meat products. For example, processed meat is often cured or salted with nitrates or nitrites; these can be converted into nitrosamines in the stomach and may have a toxic effect on the beta cells in the pancreas and impair insulin response^23^. In addition, cooking meat may produce high amounts of advanced glycation end-products^24^, which have been associated with insulin resistance^24,25^. Other potential mechanisms include the relatively high levels of saturated fatty acids and iron in meat, which have both been associated with increased insulin resistance^26–28^. Observational studies and one Mendelian randomisation study in China have suggested an association of ferritin with diabetes^29^.

The lower risk for diabetes in the low and non-meat consumers might also be related to their higher consumption of plant foods including pulses, nuts, fruits, and vegetables; we found that plant proteins were inversely associated with diabetes risk. These foods have been linked to lower fasting glucose levels, lower insulin concentration, and increased insulin sensitivity, likely due to their relatively low glycaemic load and high fibre content^30,31^. We also found that cheese consumption was higher in lower and non-meat eaters and cheese consumption has previously been associated with a lower diabetes risk^32^, possibly related to the calcium^33^, vitamin D^34^, magnesium content^35^, probiotic bacteria, and/or fatty acid composition^36^.

One explanation for fish eaters having the lowest risk might relate to BMI, because fish eaters gained the least weight over 15 years of follow-up. However, it is also possible that some of the apparent protection in the fish eaters might be attributed to their fish consumption^37^. Omega-3 polyunsaturated fatty acids may increase insulin sensitivity by improving cell membrane fluidity^38^ and help decrease insulin resistance through their anti-inflammatory effects^39^. However, the epidemiological evidence supporting the association between fish consumption and diabetes risk is inconsistent^39^. Moreover, given the relatively smaller number of fish-but-not-meat consumers and subsequently small number in this group who developed diabetes in both the current study and the AHS-2 study, the results for fish eaters should be interpreted with caution.

The important strengths of this study include the large proportion of low and non-meat eaters, the high adherence to diet group over time^40^, the long duration of follow-up, and the use of health record linkage to confirm diabetes status. However, a number of potential limitations need to be considered when interpreting our findings. The generalisability of our results may be limited by the structure of our cohort, which is predominantly of white, European descent. Also, due to the use of hospital records for case ascertainment, there may be incomplete ascertainment of participants who have existing, well-controlled diabetes, along with an over-representation of participants diagnosed with severe diabetes and related and non-related co-morbidities. Moreover, for hospitalisations or deaths not due primarily to diabetes, hospital admissions may relate to factors unrelated to diabetes and time to event might not completely align with the time of diabetes diagnoses. However, this is unlikely to relate strongly to vegetarian status. Also, we were unable to take into account diabetes medication and this may relate to dietary intake and hospitalisation. An additional consideration is that low and no-meat consumption may be an overall marker of a healthier lifestyle, and although we have adjusted the risk estimates for smoking, alcohol consumption, and physical activity, it is possible that the protective association of these diets may be partly attributed to residual confounding. Furthermore, information regarding family history of diabetes was not ascertained; therefore, we were unable to assess the potential effect of family history on the association between vegetarianism and diabetes risk.

## Conclusions

We found that people consuming a low or meat-free diet had a lower risk of hospitalisation or death with diabetes, and that this was at least partly attributable to these diet groups having a lower BMI than regular meat eaters. Further research is needed to examine the role of low meat and non-meat diets in other ethnic groups, and to determine (using genetic variants for example) whether meat is causally related to the development of diabetes.

## Supplementary information

Supplemental material
