# Supplementary material for: Vegetarian diets and risk of hospitalisation or death with diabetes in British adults: results from the EPIC-Oxford study
Source: Nutr Diabetes. 2019 Feb 25;9:7. doi: 10.1038/s41387-019-0074-0 (PMC6389979; doi:10.1038/s41387-019-0074-0)
Supplement: Supplementary file 1 — Supplemental material [file 41387_2019_74_MOESM1_ESM.docx]

EPIC-Oxford participants recruited at baseline

(1993-2001) **N=65 411**

**Excluded (n=3 150):**

-Had incomplete dietary data (n=1 293)

-Unknown body mass index (n=1 630)

-Unknown smoking status (n=227)

**Excluded (n=7 926):**

-Prevalent malignant cancers (n=1 967)

-Prevalent diabetes (n=5 303)

-Prevalent heart attack (n=476)

-Prevalent stroke (n=180)

Participants free of prevalent malignant cancers, diabetes, heart attack, or stroke

**n=48 464**

Participants with complete dietary data, and known values for body mass index and smoking status

**Analysed cohort=45 314**

Participants living in England, Scotland, or Wales who could be traced by the NHS

**n=64 432**

**N=87,151**(100%)

**Excluded (n=979):**

-Outside England, Scotland, Wales (n=945)

-Not traced by the NHS (n=34)

**Excluded (n=8 042):**

-Only completed the short survey (n=7 619)

-Aged <20 or >90 at recruitment (n=59)

-Had no follow-up data (n=364)

Participants who completed the full questionnaire, were aged >20-<90 years at recruitment, and had follow-up data

**n=56 390**

**Supplemental Figure 1: Participant flow chart for the selection of 45 314 British adults in the EPIC-Oxford study.**

**Supplemental Table 1**. Associations between five diet groups and diabetes incidence (or death due to diabetes) in 45 314 EPIC-Oxford participants

| Dietary groups | cases / total at risk | Hazard ratios, 95% Confidence intervals | |
| --- | --- | --- | --- |
|  |  | Model 1 | Model 2 |
| Regular meat eaters | 691/15181 | 1 | 1 |
| Low meat eaters | 184/7615 | 0.63 (0.54, 0.75) | 0.78 (0.66, 0.92) |
| Fish eaters | 93/7092 | 0.47 (0.38, 0.59) | 0.64 (0.51, 0.80) |
| Vegetarians | 230/13645 | 0.65 (0.55, 0.76) | 0.89 (0.75, 1.05) |
| Vegans | 26/1781 | 0.53 (0.36, 0.79) | 0.99 (0.66, 1.48) |
| *P*-value^1^ | - | <0.001 | <0.001 |

Regular meat eaters were defined as participants who consumed >50 grams of meat per day and low meat eaters were defined as participants who consumed <50 grams of meat per day. Model 1: Cox regression analysis stratified by sex, method of recruitment, region of residence and adjusted for age, education, Townsend deprivation index, ethnicity, smoking, alcohol intake, physical activity. Model 2: Cox regression analysis further adjusted for body mass index. ^1^ Represents significant heterogeneity in risk between diet groups based on likelihood-ratio test statistics.

**Supplemental Table 2.** Diabetes risk (or death) by sex-specific quintile of nutrient intakes

| **Hazard ratio and 95% CI of diabetes by sex-specific quintile of nutrient intake** | | | | | | |
| --- | --- | --- | --- | --- | --- | --- |
| Q1 Q2  n=9 064 n=9 062 | | | Q3  n=9 064 | Q4  n=9 062 | Q5  n=9 062 | *P trend*^1^ |
| Energy (kJ/day) | 1 | 0.99 (0.82, 1.19) | 1.08 (0.90, 1.29) | 1.07 (0.89, 1.28) | 1.03 (0.86, 1.24) | 0.61 |
|  |  |  |  |  |  |  |
| Carbohydrates (%E) | 1 | 0.86 (0.72, 1.02) | 0.87 (0.73, 1.04) | 0.88(0.73, 1.06) | 0.96 (0.79, 1.16) | 0.70 |
| Total sugars(%E) | 1 | 0.89 (0.75, 1.07) | 0.84 (0.70, 1.01) | 0.85 (0.71, 1.02) | 0.90 (0.75, 1.08) | 0.30 |
| Added sugars(%E) | 1 | 1.14 (0.94, 1.38) | 1.08 (0.89, 1.31) | 1.28 (1.06, 1.55) | 1.38 (1.14, 1.67) | **<0.001** |
| Intrinsic sugars(%E) | 1 | 0.84 (0.71, 1.00) | 0.79 (0.67, 0.95) | 0.71 (0.60, 0.86) | 0.71 (0.58, 0.85) | **<0.001** |
| Starch (%E) | 1 | 0.97 (0.83, 1.14) | 0.78 (0.66, 0.94) | 0.87 (0.72, 1.04) | 1.11 (0.92, 1.33) | 0.80 |
|  |  |  |  |  |  |  |
| Fibre (g)* | 1 | 1.04 (0.88, 1.24) | 0.99 (0.83, 1.18) | 0.93 (0.78, 1.11) | 0.81 (0.67, 0.99) | **0.02** |
|  |  |  |  |  |  |  |
| Protein (%E) | 1 | 0.99 (0.80, 1.24) | 0.92 (0.75, 1.14) | 1.09 (0.89, 1.33) | 1.03 (0.85, 1.27) | 0.46 |
| Animal protein (%E) | 1 | 0.81 (0.64, 1.02) | 0.86 (0.69, 1.08) | 1.03 (0.84, 1.27) | 1.02 (0.83, 1.25) | 0.12 |
| Plant protein (%E) | 1 | 0.78 (0.67, 0.92) | 0.73 (0.61, 0.86) | 0.72 (0.61, 0.87) | 0.72 (0.59, 0.88) | **<0.001** |
|  |  |  |  |  |  |  |
| Fat (%E) | 1 | 1.01 (0.84, 1.21) | 0.89 (0.74, 1.07) | 1.01 (0.84, 1.22) | 1.02 (0.85, 1.23) | 0.82 |
| SFA (%E) | 1 | 0.95 (0.79, 1.15) | 0.86 (0.71, 1.04) | 1.02 (0.85, 1.23) | 1.05 (0.87, 1.26) | 0.34 |
| MUFA (%E) | 1 | 0.82 (0.68, 0.99) | 0.83 (0.69, 1.00) | 0.94 (0.78, 1.13) | 1.01 (0.84, 1.21) | 0.46 |
| PUFA (%E) | 1 | 1.10 (0.93, 1.30) | 0.94 (0.79, 1.12) | 1.03 (0.86, 1.23) | 0.99 (0.82, 1.20) | 0.75 |
| EPA (%E) | 1 | 0.75 (0.60, 0.94) | 0.89 (0.73, 1.09) | 0.95 (0.78, 1.16) | 0.89 (0.73, 1.09) | 0.87 |
| DHA (%E) | 1 | 0.81 (0.64, 1.01) | 0.89 (0.72, 1.10) | 1.00 (0.81, 1.23) | 0.89 (0.72, 1.10) | 0.88 |

*quintile numbers differ slightly. SFA, saturated fatty acid; MUFA, monounsaturated fatty acid; PUFA, polyunsaturated fatty acid; EPA, eicosapentaenoic acid; DHA, docosahexaenoic acid. Cox regression analysis stratified by sex, method of recruitment and region of residence and adjusted for age, education, Townsend index, smoke, alcohol, physical activity, BMI, and energy (*energy and fibre not adjusted for energy). ^1^ Tests for trend were assessed by replacing the categorical variable with a continuous variable containing the sex-specific medians for each quintile.
